# Supplementary material for: What Is a School Farm? Results of a Scoping Review
Source: Int J Environ Res Public Health. 2023 Mar 30;20(7):5332. doi: 10.3390/ijerph20075332 (PMC10094287; doi:10.3390/ijerph20075332)
Supplement: Supplementary file 1 [file ijerph-20-05332-s001.zip › ijerph-2250553-supplementary.pdf]

Supplementary Table S1. Summary of included sources in scoping review of school farm literature.

Author/s	Title	Year of Publication	Article Type	Country/ Region	Age/Grade Demographics	Sample Size	Gender	Geographic Size
NR	New school farm for Boise.	1916	Editorial	US	Secondary school students	NR	NR	40 Acres
NR	Boise's progress.	1917	Editorial	USA	NR	NR	Boys	40 Acres Divided Into 8 5-Acre Tracts
Day	The place of the school farm in a secondary vocational agricultural instruction.	1921	Editorial	USA	NR	NR	Boys	NR
NR	School farmers make neat profit	1934	Editorial	USA	NR	262 students	NR	NR
NR	School farms in India	1938	Editorial	India	Primary and secondary school students	NR	Boys	NR
Cross	Why a school farm?	1939	Editorial	USA	School-aged* FFA members	NR	Boys	10–50-Acre Plots
Dickson	Use of a school farm as a laboratory for vocational agriculture.	1939	Editorial	USA	NR	About 300 students	Boys	100 Acres
Balloun	Using a school farm in teaching.	1939	Editorial	USA	School-aged* FFA members	NR	Boys	15 Acres
Vazquez-Torres	Directed practice in the school farms of Puerto Rico.	1939	Editorial	Puerto Rico	School-aged students*	NR	Boys	5 Acres or Larger
NR	School farm in the Transvaal: Lord Milner School Farm.	1943	Editorial	South Africa	Primary school students	NR	Boys & Girls	NR
Pack	Students in Hawaii produce food on the school farm.	1943	Editorial	USA	Secondary school students	NR	Boys	1 Acre
NR	School farms.	1944	Editorial	UK	School-aged students*	NR	Boys	N/A

Ako	Relation of the school farm to the instructional program in Hawaii.	1945	Editorial	USA	School-aged students*	NR	Boys	2-Acre Vegetable Plot
Johnson	School farm and plots in the Pacific region.	1947	Editorial	USA	NR	NR	Boys	32.9 Acres-Owned And 57.8-Leased (Averages)
Bressler	We moved our classes to the farm.	1948	Editorial	USA	Secondary school students & veterans	NR	Boys	750 Acres Divided In 3 Sections.
Mcmahon & Mcmahan	Conservation at the Battle Creek Public School Farm	1949	Editorial	USA	School-aged students*	150 students	Boys & Girls	NR
Brown	School farm becomes much used resource.	1949	Editorial	USA	NR	NR	Boys	51 Acres
Eckelberry	School farm offers educational opportunity.	1949	Editorial	USA	Secondary school students	50% of students in the school	Boys	121 Acres
Spearin	Cooperative school farm.	1950	Editorial	USA	NR	NR	Boys	15 Acres
Ahalt	School farms in the North Atlantic region.	1951	Editorial	USA	Secondary school students	NR	Boys	3–750-Acre Plots
Clark	Observations on use of school farms in Michigan.	1951	Editorial	USA	Mostly secondary school students	NR	Boys	120 -160 Acres
Hutton	West Virginia's school Farm.	1951	Editorial	USA	Secondary school students	NR	Boys	NR
Mcdonald	School farms In Maryland.	1951	Editorial	USA	School-aged students*	NR	Boys	3-40 Acres.
Cazaly	High school farm operated as a commercial unit.	1951	Editorial	USA	Secondary school students	NR	Boys	80 Acres
Jensen	Establishment and organization of the Visalia school farm.	1951	Editorial	USA	Secondary school students & junior college** students	175 secondary school students, More than 100 junior college students	Boys	40 Acres

Hagenbuch & Brannaka	Quakertown High School Farm.	1952	Editorial	USA	Secondary school students	NR	Boys	100 Acres
Spilsbury	School farm laboratory supplements classroom teaching.	1952	Editorial	USA	Secondary school students	NR	Boys	95 Acres
	Harrow school farm.	1953	Editorial	UK	School-aged Students*	NR	Boys	155 Acres
Welch	School farm as a training center.	1953	Editorial	USA	Secondary school students	NR	Boys	65 Acres
Crandall	Favorable learning opportunity on a school farm.	1953	Editorial	USA	Secondary school students	NR	Boys	50 Acres
Juergenson	Community is your school farm.	1953	Editorial	USA	Secondary school students	NR	Boys	3-300 Acres
Kabler	School farms can be valuable.	1954	Editorial	USA	Secondary school students	50-80 students	Boys	40-45 Acres
Sherman	Share agreement as a means of stocking school farms	1955	Editorial	USA	Junior college** school farm	NR	Boys	300 Acres
Snell	School farms and group farming enterprises.	1955	Editorial	USA	Secondary school students	NR	Boys	NR
Sherman	School farm has public relations value.	1956	Editorial	USA	Post-secondary students	NR	NR	400 Acres
Smith	Place of a school farm.	1956	Editorial	USA	School-aged Students*	NR	Boys	93 Acres
Garvie	School farm	1957	Editorial	USA	Primary students (Grades 5 & 6)	90 students	NR	12 Acres
Booska	School farm in adjusting local program.	1960	Editorial	USA	NR	NR	NR	NR
Ballard	School farm.	1960	Editorial	USA	School-aged Students*	NR	Boys	NR
Nicklas	How the school farm operates at the U. Of Nebraska School of Agriculture.	1960	Editorial	USA	Secondary school students	NR	Boys	450 Acres
Bryant	Developing A school farm.	1960	Editorial	USA	NR	NR	NR	140 Acres
Hohman	Doing to learn, on the school farm.	1961	Editorial	USA	NR	NR	Boys	60 Acres
Haight	Using the school farm effectively.	1961	Editorial	USA	NR	NR	Boys	235 Acres

Duff	Do you need a school farm?	1970	Editorial	USA	Secondary school students	NR	Boys	3-300 Acres
Bearden	Morris FFA School Farm provides challenge.	1971	Editorial	USA	NR	29 students	Boys	10 Acres
Orhwall	School farms and country parks.	1972	Editorial	USA	Secondary school students	NR	Boys	245 Acres
Hammer	Using a school farm.	1974	Editorial	USA	Secondary school students	NR	NR	70 Acres
Mabee	Report to the government of Malaysia, on programme of the Agricultural Institutes of the Ministry of Agriculture and Fisheries, West Malaysia	1974	Government Report	Malaysia	NR	NR	NR	NR
Mcmillion	School farm In 1975.	1975	Editorial	NR	Secondary school students	NR	NR	NR
Stump	Conservation studies down on the school farm.	1976	Editorial	USA	Secondary school students	NR	NR	230 Acres
Puckett	School farm.	1977	Editorial	USA	NR	NR	NR	108 Acres
Alcock	Mdukatshani – experiment in agricultural development.	1977	Original study	South Africa	Any farmers or students interested	NR	Boys & Girls	61.78 Acres
Udo	Land laboratory use in teaching agriculture in the southern United States with implications for agricultural education in Nigeria.	1979	Dissertation	Nigeria And USA	Secondary school teachers and students	US: 69 vocational agriculture teachers from 69 public high schools; Nigeria: 26 agricultural science teachers from 26 high schools	Boys & Girls	620 Acres
Adams	Challenge of establishing a school farm.	1980	Editorial	USA	Secondary school students (Grades 11 and 12)	20 students	NR	190 Acres

Farrell	The school farm: educating through laboratory experience.	1983	Editorial	USA	Secondary school students & alumni	NR	NR	160 Acres
Stump	Prairie Heights School Farm: SOEP in conservation.	1984	Editorial	USA	NR	NR	NR	230 Acres
Olaitan	Agricultural education in the tropics. Methodology for teaching agriculture.	1984	Textbook	International	NR	NR	Boys & Girls	NR
Williams & Mccarthy	Student benefits from school farm activities as perceived by administrators and instructors.	1985	Original study	USA (4 states)	Vocational agriculture departments for school-aged students*	68 departments	Boys & Girls	10 Acres or Less
Martin & Dormody	Zuni School Farm: A bridge between vocational and academic education.	1992	Editorial	USA (Zuni Pueblo)	Secondary school students	NR	Boys	25 Acres
Martin & Dormody	Dreams becoming realities: The Zuni School Farm Project.	1994	Editorial	USA (Zuni Pueblo)	Secondary school students	NR	Boys	25 Acres
Mcgavin	Extra milk.	1995	Editorial	UK	School-aged students*	40 students	Boys & Girls	NR
Konoshima	Participation of school children in agricultural activities at school farms in Shiga prefecture.	1995	Original study	Japan	Kindergarten and primary school students	NR	Boys & Girls	NR
Thomas	Barn storming.	1996	Editorial	UK	Secondary school students	NR	Boys & Girls	2.5 Acres
Haigh	Dig in to pastures new.	1997	Editorial	UK	NR	NR	Boys	NR
Newnham	Cows on the curriculum.	2000	Editorial	UK	School farms for school-aged students*	111 school farms	Boys & Girls	44.94 Acres
Brown	Cows the classroom: A school farm for inclusive education, general farm maintenance, construction, gardening, landcare projects on farm and in the community	2001	Editorial	Australia (Tasmania)	Primary school students	NR	NR	8 Hectares

Mcgavin	Learning 'til the cows come home.	2002	Editorial	UK	Secondary school students	220 students	NR	140 Acres
Swan	Solving problems through action research: Engaging the teacher and student through exploratory learning.	2004	Editorial	US	NR	NR	NR	20 Acres
Parkin	The daily rind.	2005	Editorial	UK	Community college students	1,400 students	NR	4.94-49.42 Acres
Foeken et al.	School farming and school feeding in Nakuru Town, Kenya: Practice and potential	2007	Other (Working paper)	Kenya	Primary & secondary schools	116 schools	NR	0.1-5 Acres
Morrison	Best baa none.	2008	Editorial	UK	Primary schools	NR	NR	NR
Marley	Farm school keeps lads on the right track.	2009	Editorial	UK	13–15-Year-Olds	175 students	Boys	NR
Foeken et al..	Coping with increasing food prices in Nakuru, Kenya: Urban school farming as a way to make school lunches affordable.	2009	News	Kenya	NR	750 Students	NR	0.1 -5 Acres
Foeken et al.	Urban school farming to improve school feeding: The case of Nakuru Town, Kenya.	2010	Original study	Kenya	Primary & secondary schools	116 schools	NR	0.1 -5 Acres
Sayre & Clark	Fields of learning: The student farm movement in North America	2011	Textbook	North America	Post-secondary students	NR	Boys & Girls	NR
Warsh	Cultivating citizens: The Children's School Farm in New York City, 1902-1931	2011	Original study	USA	Secondary school students	NR	Boys & Girls	7 Acres
Wydler	The state of the art of school farming in Switzerland - The case Of Schub.	2012	Original study	Switzerland	Primary school students (grades 1 to 9)	28,000 students	NR	NR
Tearle	Where there's muck…	2013	Editorial	UK	3 - 16-year-olds with moderate to severe learning/emotional/social/behavioral difficulties	NR	NR	5.5 Acres
Summers	Tomatoes, cucumbers, and salad tag: A farmer goes to school.	2013	Editorial	USA	primary and secondary schools	5 schools	Boys	.5 Acre

Jenkins	Crops to classrooms: How school farm are growing student engagement	2014	Editorial	UK	NR	100+ school farms	NR	3 Acres
Walters	At the Table.	2014	Editorial	USA	NR		NR	NR
Carten	Farming school grounds.	2014	Editorial	Canada	Primary and secondary students in Toronto and Vancouver	Toronto, 14 students Vancouver, 30-4-students	NR	NR
NA	Lancing Farm Project, 30 Years On.	2014	Editorial	USA	NR	NR	NR	NR
Green Schools National Network	Denver Public Schools Farm to School Program: School farms feed district students.	2015	News	USA	NR	NR	NR	NR
Paffarini et al.	Bridging the Gap - Education and in specialized kindergarten prgorams	2015	Original study	Europe	Children from 2 kindergarten schools	Italy, 4 students Germany, 20 students	NR	Italy: 123.55 Acres Germany: 61.78 Acres
Ambroise et al.	Perfea: Ongoing counselling towards strategic planning processes to implement the agro-ecological transition	2016	Other (Conference Paper)	France	Secondary schools	190 School Farms, 33 Technical Processing Plants	Boys & Girls	NR
Grambril	School farm safety: Avoiding or being the headlines.	2016	Editorial	USA	Secondary schools	NR	NR	NR
Aniebiat Okon	Strategies for school farmland conflict resolution and effective teaching and research in agricultural education	2017	Original study	Nigeria	NR	70 Agricultural teachers, 20 School management staff, 60 community leaders in school communities	NR	NR
Twenter & Edwards	Facilities in school-based, agricultural education (SBAE): A historical inquiry	2017	Original study	USA	Primary & secondary school students	NR	Boys	5-300 Acres

Corbett et al.	What we're about out here: The resilience and relevance of school arm in rural Tasmania.	2017	Original study	Australia (Tasmania)	Primary and secondary schools	22 School Farm Educators	Boys	49.42 Acres
Fifolt et al.	Promoting school connectedness among minority youth through experience-based urban farming	2018	Original study	USA	Kindergarten, primary, and secondary school students	33 students, 25 parents	Boys	2-40 Acres
Yopp et al.	Flipped programs: Traditional agricultural education in non-traditional programs.	2018	Original study	USA	Secondary school agricultural programs	3 agricultural programs, 3 teachers	Boys	NR
Lambert et al.	Understanding characteristics, uses, perceptions, and barriers related to school farms in Oregon.	2018	Original study	USA	Oregon secondary school agricultural education teachers	64 teachers	Boys	1-60 Acres
Fifolt & Morgan	Engaging K-8 students through inquiry-based learning and school farms.	2019	Original study	USA	Primary school staff	15 Teachers, 5 Principals	NR	NR

Abbreviations: TES, Times Education Supplement; AEM, Agricultural Education Magazine; NR, Not Reported. * School-aged students were undefined, so students could be in any grade from kindergarten to high school. **Junior colleges are 2-year post-secondary schools common in the United States.
